# Supplementary material for: Prefusion-stabilized Hantaan virus glycoprotein nucleic acid vaccine elicits potent neutralizing antibody responses via germinal center activation
Source: Nat Commun. 2026 Mar 14;17:3972. doi: 10.1038/s41467-026-70285-7 (PMC13133349; doi:10.1038/s41467-026-70285-7)
Supplement: Supplementary file 1 — Supplementary Information [file 41467_2026_70285_MOESM1_ESM.pdf]

Supplemental Information

Prefusion-stabilized Hantaan Virus Glycoprotein Nucleic Acid Vaccine Elicits Potent Neutralizing Antibody Responses via Germinal Center Activation

Wei Ye (叶伟) [1, †, *], Yamei Dang (党亚美) [1, †,] Yuan Wang (王媛) [1, †], Qiqi Yang (杨淇淇) [1, †], Hui Zhang (张惠) [1, †], Chuantao Ye (叶传涛)[2, †], Jing Wei (魏菁) [1,3, †], Jiawei Pei (裴佳伟)[1], Xuemin Pei (裴雪敏)[1,4], Dongshen Jiang (姜东绅) [1,5], Xiaojing Yang (杨晓静)[1,5], Xiaolei Jin (靳晓磊) [6], Hongwei Ma (马宏炜) [1], He Liu (刘赫)[1], Liang Zhang (张亮) [1], Linfeng Cheng (程林峰) [1], Yangchao Dong (董阳超)[1], Yingfeng Lei (雷迎峰) [1, *], Zhikai Xu (徐志凯)[1, *], Fanglin Zhang (张芳琳)[1, *]

[1] Department of Microbiology, School of Preclinical Medicine, Airforce Medical University: Fourth Military Medical University, Xi'an, Shaanxi 710032, China

[2] Department of Infectious Diseases, Tangdu Hospital, Airforce Medical University: Fourth Military Medical University, Xi'an, Shaanxi 710038, China

[3] Center for Disease Control and Prevention of Shaanxi Province, Xi'an, Shaanxi 710054, China.

[4] School of Medicine, Northwest University, Xi'an, Shaanxi, 710069, China.

[5] School of Medicine, Yan'an University, Yan'an, Shaanxi 716000, China.

[6] Student Brigade, School of Preclinical Medicine, Airforce Medical University: Fourth Military Medical University, Xi'an, Shaanxi, 710032, China.

[7] These authors contributed equally to this work.

[8] Correspondence: Wei Ye, virologyyw@fmmu.edu.cn; Yingfeng Lei, yflei@ fmmu.edu.cn; Zhikai Xu, zhikaixu@fmmu.edu.cn; Fanglin Zhang, flzhang@fmmu.edu.cn;

Supplemental Fig. 1 Design, characterization, and preliminary immunogenicity of prefusion-stabilized HTNV GP antigens. (**Related to Fig. 1**)

Supplemental Fig. 2. T-cell responses induced by prefusion-stabilized HTNV GP DNA vaccines. (**Related to Fig. 1**)

Supplemental Fig. 3. Protective efficacy of HTNV GP DNA vaccine in challenged mice. (**Related to Fig. 2**)

Supplemental Fig. 4. Long-term antigen-specific T-cell responses induced by HTNV GP DNA vaccines. (**Related to Fig. 3**)

Supplemental Fig. 5. Gating strategy for identifying T follicular helper and germinal center B cells. (**Related to Fig. 4**)

Supplemental Fig. 6. Characterization of HTNV GP mRNA-LNP and vaccine-induced T-cell responses. (**Related to Fig. 5**)

Supplemental Fig. 7. Protective efficacy of HTNV GP mRNA vaccine in challenged mice. (**Related to Fig. 6**)

Supplemental Fig. 8. Booster immunization enhances T-cell immunity. (**Related to Fig. 8**)

Supplemental Table 1. Comparative immunogenicity and efficacy of the prefusion-stabilized GP-C3 vaccine delivered via DNA or mRNA-LNP platforms.

Supplemental Table 2. HTNV Gn 15-mer peptides with an 8-amino acid overlap.

Supplemental Table 3. HTNV Gc 15-mer peptides with an 8-amino acid overlap.

Supplemental Fig. 1

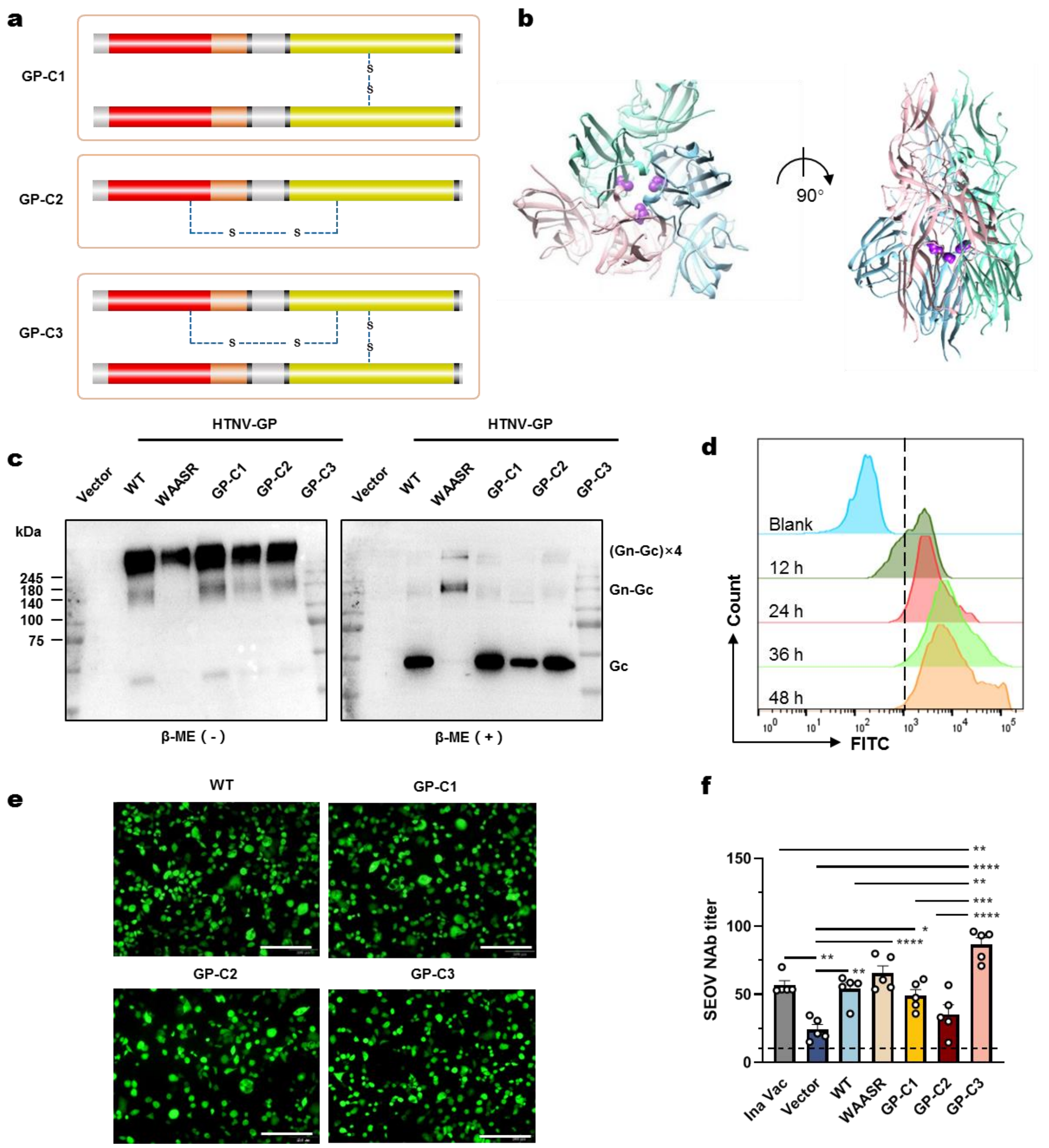

Supplemental Fig. 1 Design, characterization, and preliminary immunogenicity of prefusion-stabilized HTNV GP antigens.

(a) Schematic diagram of the GP-C1, GP-C2, and GP-C3 immunogens. **(b)** Model of the HTNV post-fusion Gc trimer (PDB: 5LJZ) was constructed using SWISS-MODEL and

visualized in Chimera, with residue G835 highlighted in violet. **(c)** Lysates from HEK-293T cells transfected with pVAX1 vectors expressing different HTNV GP constructs or empty vector were analyzed by Native PAGE (left) and reducing SDS-PAGE (right). **(d)** Representative flow cytometry histograms of GFP expression at indicated times post-transfection. **(e)** HEK-293T cells were co-transfected with HTNV GP, ITGB3, and GFP plasmids. At 24 h post-transfection, cells were treated with citric acid (pH 5.0) to induce membrane fusion, and syncytia were counted. Scale bar, 200 μm. **(f)** Seoul virus (SEOV)-neutralizing antibody (NAb) titers in mouse sera at day 14 post-immunization. Data are mean ± SEM (n = 5). Significance was determined by one-way ANOVA with Tukey's test. *P < 0.05, **P < 0.01, ***P < 0.001, ****P < 0.0001. Exact P values are provided in the Source Data file. Source data are provided as a Source Data file.

Related to Fig. 1

Supplemental Fig. 2.

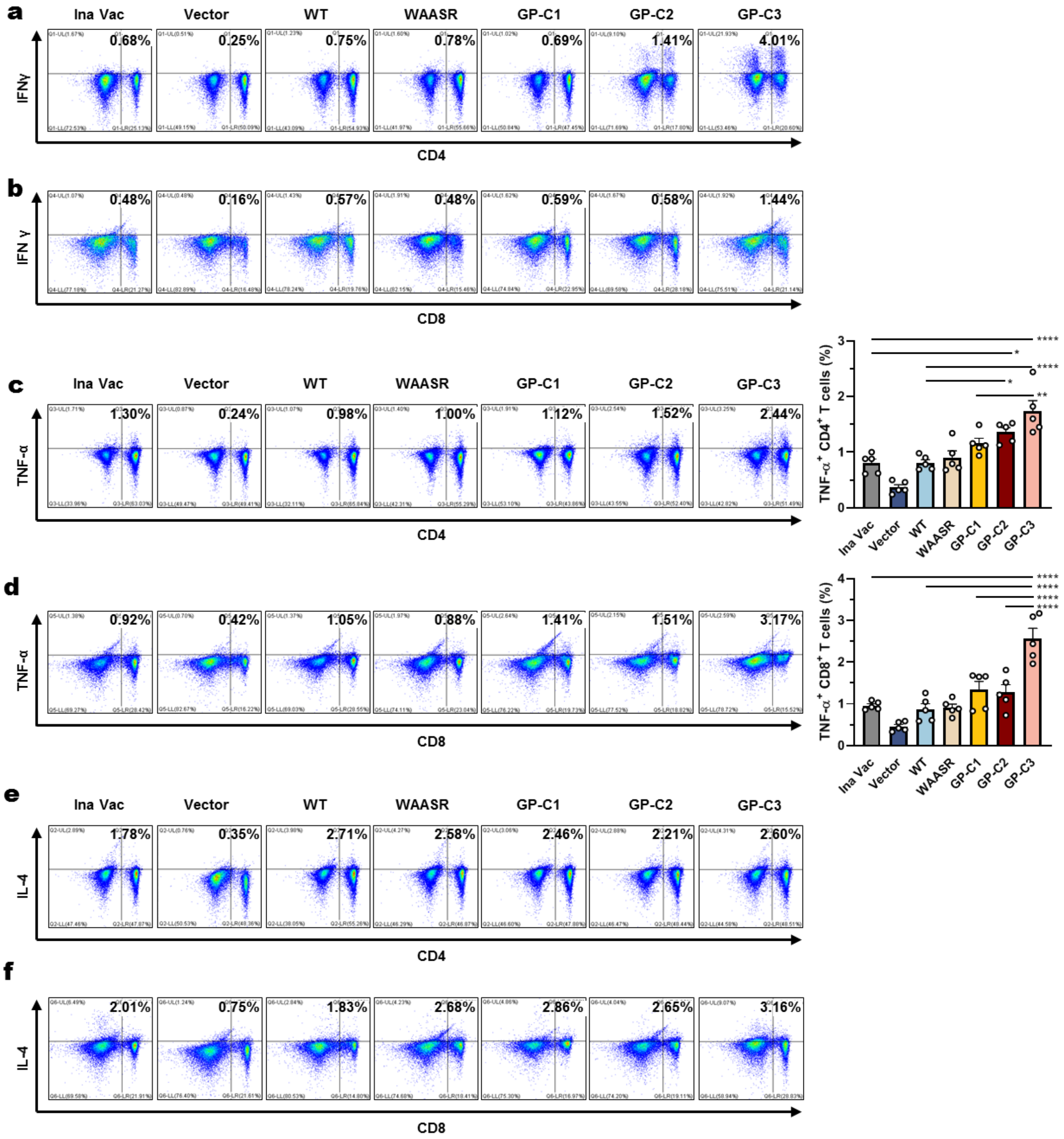

Supplemental Fig. 2. T-cell responses induced by prefusion-stabilized HTNV GP DNA vaccines.

(a, b) Quantification of IFN-γ$^+$ CD4$^+$ (a) and CD8$^+$ T cells (b) in response to HTNV-GP peptides.

(c, d) Analysis of TNF-α$^+$ CD4$^+$ (c) and CD8$^+$ T cells (d). Representative flow cytometry

plots (left panels) and corresponding quantitative summaries (right panels) are shown.

(e, f) Quantification of IL-4$^+$ CD4$^+$ (e) and CD8$^+$ T cells (f).

Data are presented as mean ± SEM (n = 5). Statistical significance was determined by one-way ANOVA with Tukey's multiple comparisons test. *P < 0.05, **P < 0.01, ****P < 0.0001. Exact P values are provided in the Source Data file. Source data are provided as a Source Data file.

Related to Fig. 1

Supplemental Fig. 3.

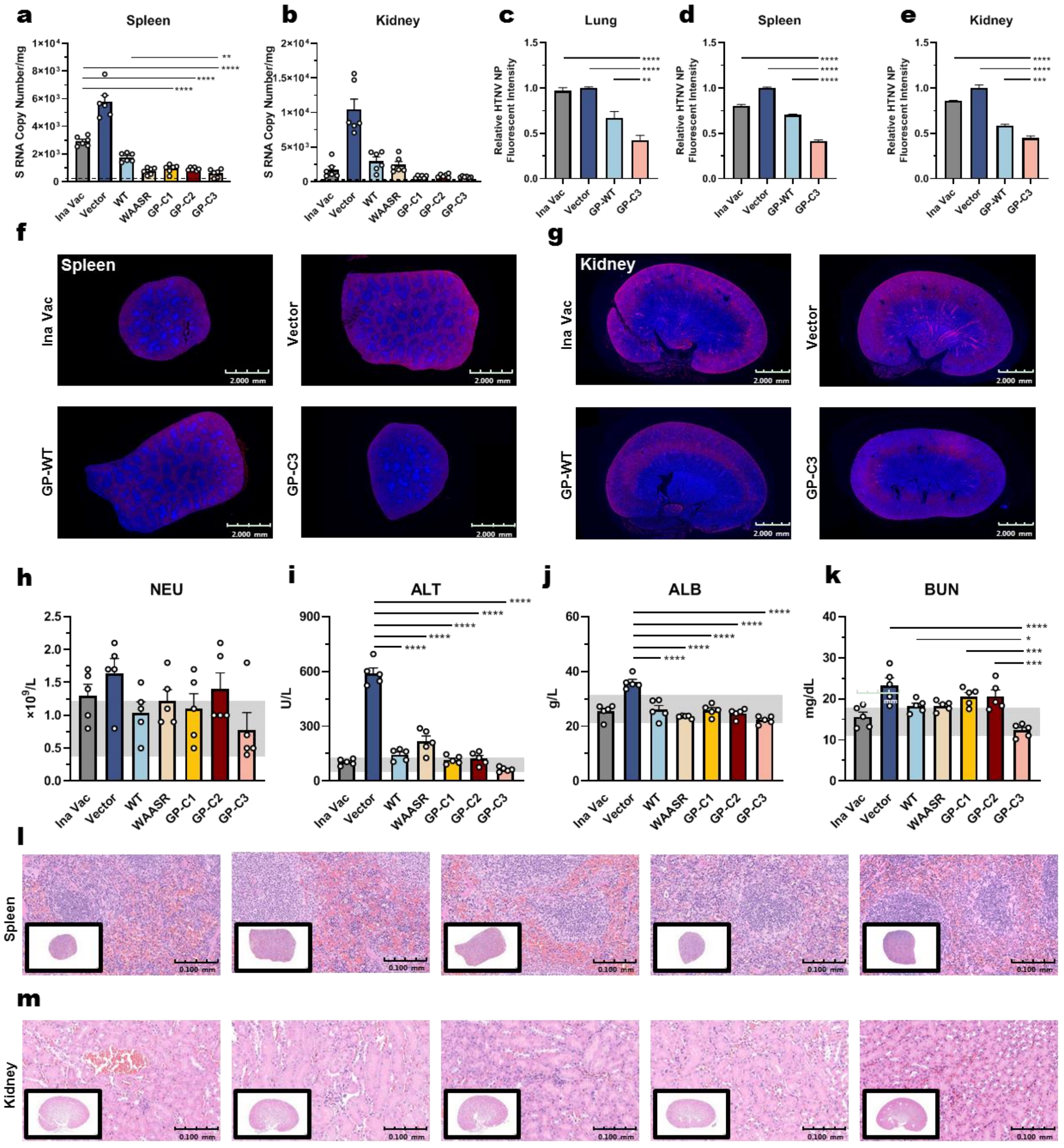

Supplemental Fig. 3. Protective efficacy of HTNV GP DNA vaccine in challenged mice.

(a, b) Viral RNA loads in spleen (a) and kidney (b) tissues measured by qRT-PCR. The dashed line indicates the detection limit. **(c-e)** Quantification of viral antigen signal by immunofluorescence in lung (c), spleen (d), and kidney (e) sections at 3 days post-infection (dpi). **(f, g)** Representative immunofluorescence images of spleen (f) and kidney

(g) at 3 dpi. Red, HTNV nucleoprotein (NP); blue, DAPI (nuclei). Scale bars, 2 mm. **(h-k)** Hematological and serum biochemical analyses at 3 dpi; neutrophil count (NEU, h), alanine aminotransferase (ALT, i), albumin (ALB, j), and blood urea nitrogen (BUN, k). **(l, m)** H&E staining of spleen (l) and kidney (m) sections at 3 dpi. Scale bars, 100 μm. Data are presented as mean ± SEM (n = 5 or 6 per group). Significance was determined by one-way ANOVA with Tukey's multiple comparisons test. *P < 0.05, **P < 0.01, ***P < 0.001, ****P < 0.0001. Exact P values are provided in the Source Data file. Source data are provided as a Source Data file.

Related to Fig. 2

Supplemental Fig. 4.

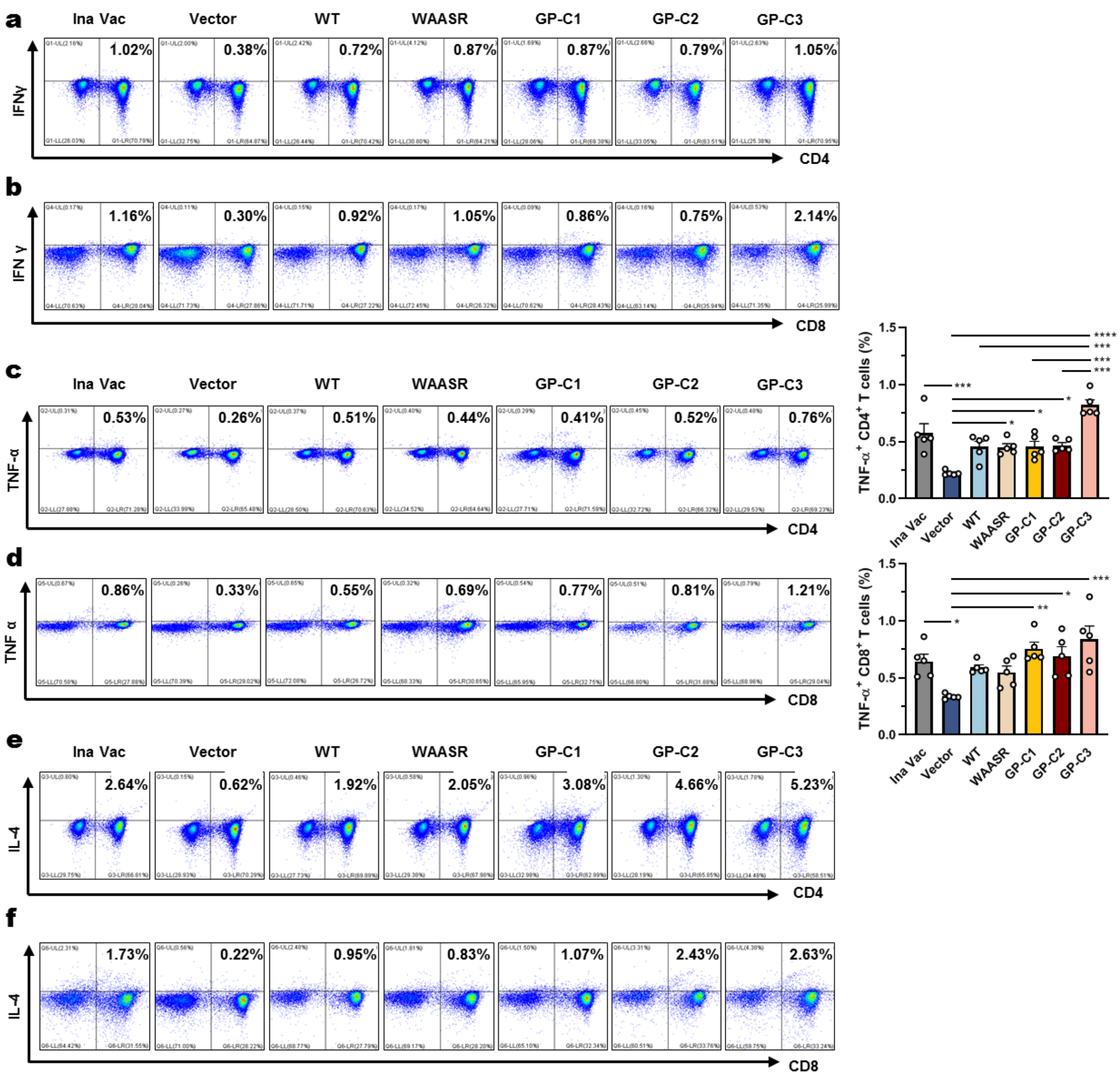

Supplemental Fig. 4. Long-term antigen-specific T-cell responses induced by HTNV GP DNA vaccines.

(a, b) Quantification of IFN-γ^{+} CD4^{+} (a) and CD8^{+} T cells (b) in response to HTNV-GP peptides at the indicated long-term time point.

(c, d) Analysis of TNF-α^{+} CD4^{+} (c) and CD8^{+} T cells (d). Representative flow cytometry plots (left panels) and corresponding quantitative summaries (right panels) are shown.

(e, f) Quantification of IL-4^{+} CD4^{+} (e) and CD8^{+} T cells (f).

Data are presented as mean ± SEM (n = 5). Statistical significance was determined by one-way ANOVA with Tukey's multiple comparisons test. *$P < 0.05$, **$P < 0.01$, ****$P < 0.0001$. Exact P values are provided in the Source Data file. Source data are provided as a Source Data file.

Related to Fig. 3

Supplemental Fig. 5.

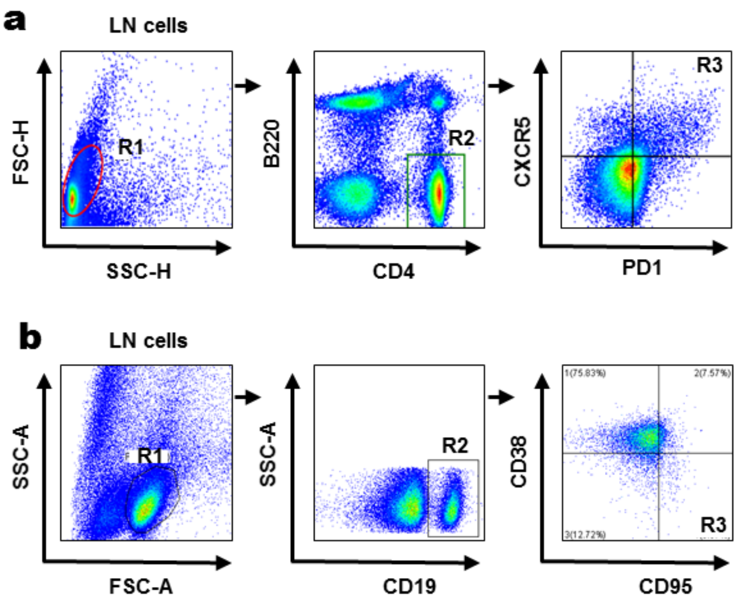

Supplemental Fig. 5. Gating strategy for identifying T follicular helper and germinal center B cells.

(a, b) Flow cytometry gating strategy used to identify immune cell populations in draining lymph nodes.

(a) Sequential gating for T follicular helper (Tfh) cells: lymphocytes (FSC-H vs. SSC-H) → single cells → $CD4^+$ T cells ($CD4^+B220^-$) → $CXCR5^+PD1^+$ Tfh cells.

(b) Sequential gating for germinal center B (GC B) cells: lymphocytes (FSC-A vs. SSC-A) → single cells → B cells ($CD19^+$) → GC B cells ($CD38^-CD95^+$).

Related to Fig. 4

Supplemental Fig. 6.

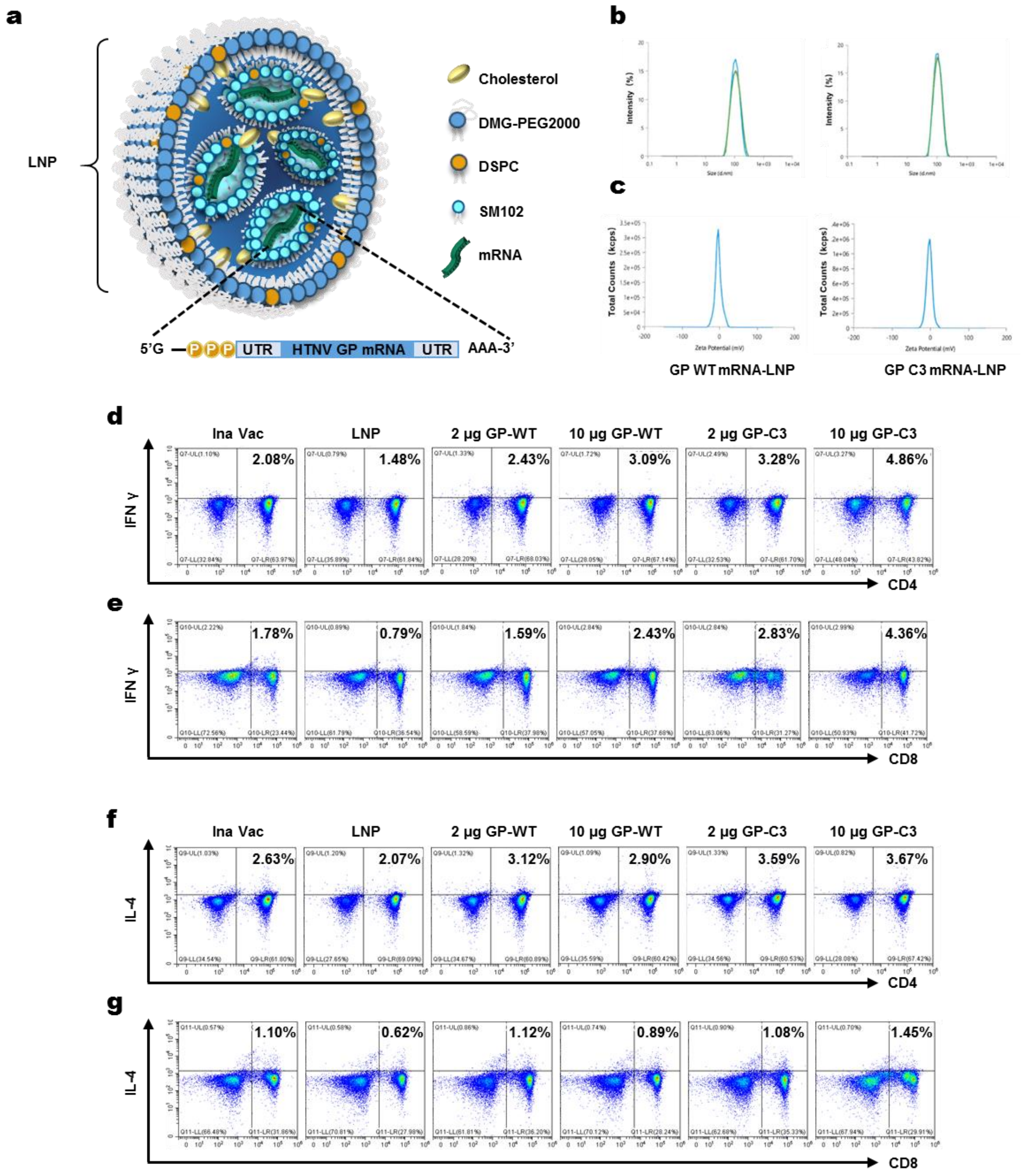

Supplemental Fig. 6. Characterization of HTNV GP mRNA-LNP and vaccine-induced T-cell responses.

(a) Schematic of the mRNA-LNP vaccine construct.

(b,c) Physicochemical characterization of mRNA-LNP particles: hydrodynamic size (b) and zeta potential (c) of GP-WT (left) and GP-C3 (right) formulations.

(d–g) Frequency of antigen-specific cytokine-producing T cells in immunized mice. Quantification of IFN-γ^+ $CD4^+$ T (d) and $CD8^+$ T (e), and IL-4^+ $CD4^+$ T (f) and $CD8^+$ T cells (g) in response to HTNV-GP peptide stimulation.

Related to Fig. 5

Supplemental Fig. 7.

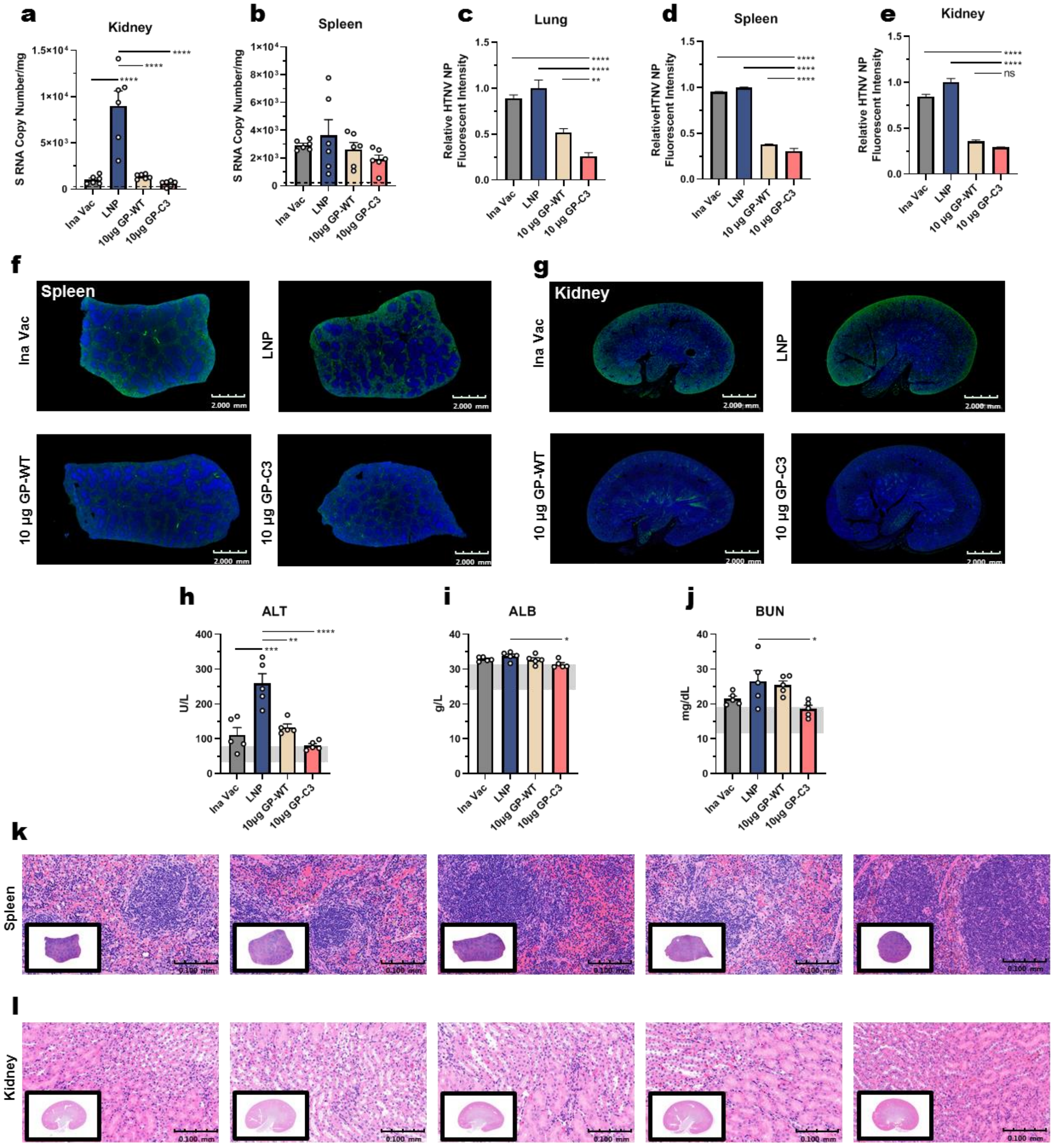

Supplemental Fig. 7. Protective efficacy of HTNV GP mRNA vaccine in challenged mice.

(a, b) Viral RNA loads in kidney (a) and spleen (b) tissues measured by qRT-PCR. The dashed line indicates the detection limit. **(c-e)** Quantification of viral antigen signal by immunofluorescence in lung (c), spleen (d), and kidney (e) sections at 3 days post-

infection (dpi). **(f, g)** Representative immunofluorescence images of spleen (f) and kidney (g) at 3 dpi. Red, HTNV nucleoprotein (NP); blue, DAPI (nuclei). Scale bars, 2 mm. **(h-j)** Hematological and serum biochemical analyses at 3 dpi; ALT (h), ALB (i), and BUN (j). **(k, l)** H&E staining of spleen (k) and kidney (l) sections at 3 dpi. Scale bars, 100 μm. Data are presented as mean ± SEM (n = 5 or 6 per group). Significance was determined by one-way ANOVA with Tukey's multiple comparisons test. *P < 0.05, **P < 0.01, ***P < 0.001, ****P < 0.0001. Exact P values are provided in the Source Data file. Source data are provided as a Source Data file.

Related to Fig. 6

Supplemental Fig. 8.

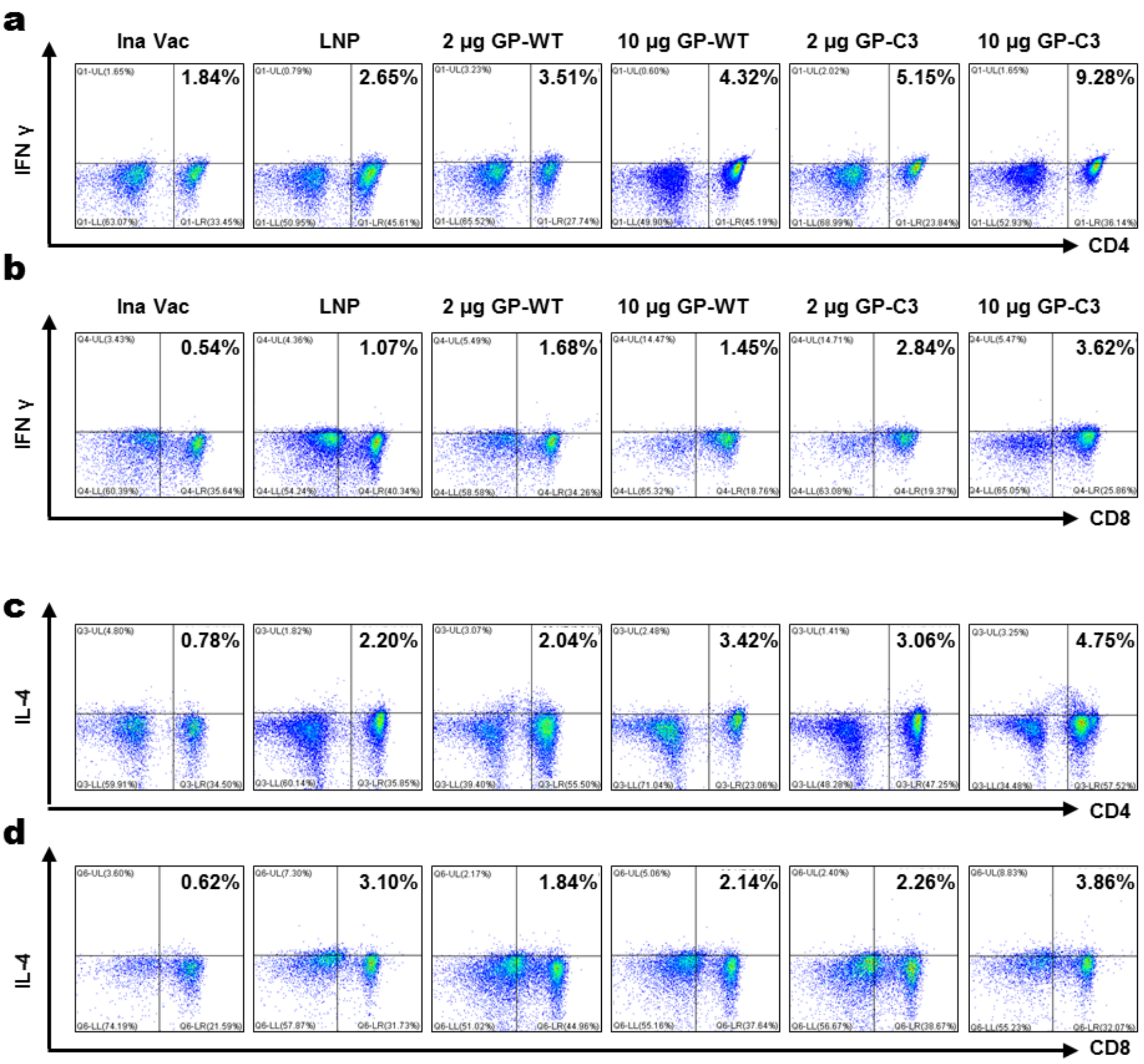

Supplemental Fig. 8. Booster immunization enhances T-cell immunity.

(a–d) Representative frequency of intracellular IFN-γ expressing $CD4^+$ T (a) and $CD8^+$ T (b), IL-4 expressing $CD4^+$ T (c), and $CD8^+$ T (d) specific for HTNV-GP peptides after the prime-boost vaccination schedule.

Related to Fig. 8

Supplemental Table 1. Comparative immunogenicity and efficacy of the prefusion-stabilized GP-C3 vaccine delivered via DNA or mRNA-LNP platforms.

DNA platform	Neutralizing Antibody Titer (GMT) (Day 70)		mRNA-LNP platforms	Neutralizing Antibody Titer (GMT) (Day 28)	
**Fig. 1g, h**	**HTNV**	**SEOV**	**Fig. 5f, g**	**HTNV**	**SEOV**
**Ina Vac**	46.44	40.54	**Ina Vac**	65.32	44.87
**Vector**	21.36	25.92	**LNP**	23.50	25.88
**WT**	62.64	49.30	**2 µg WT**	71.84	
**GP-C1**	54.93	49.54	**10 µg WT**	84.68	130.39
**GP-C2**	61.21	62.75	**2 µg GP-C3**	100.36	
**GP-C3**	170.12	94.18	**10 µg GP-C3**	247.16	242.64
	**Fold Reduction to Vector**			**Fold Reduction to LNP**	
**Fig. 2a, b**	**Lung**	**Liver**	**Fig. 5a, b**	**Lung**	**Liver**
**Ina Vac**	1.7-fold	1.6-fold	**Ina Vac**	2.1-fold	2.8-fold
**WT**	1.6-fold	2.2-fold			
**GP-C1**	6.5-fold	5.2-fold	**10 µg WT**	4.3-fold	2.7-fold
**GP-C2**	5.4-fold	8.7-fold			
**GP-C3**	10.8-fold	7.2-fold	**10 µg GP-C3**	11.6-fold	7.2-fold
	**Average Frequency (%)**			**Average Frequency (%)**	
**Fig. 4c,e**	**Tfh**	**GC B**	**Fig. 5a, b**	**Tfh**	**GC B**
**Ina Vac**	7.61	3.8	**Ina Vac**	7.80	6.22
**Vector**	1.48	1.7	**LNP**	3.19	3.85
**WT**	4.08	4.8	**10 µg WT**	10.02	6.32
**GP-C3**	14.61	9.52	**10 µg GP-C3**	26.79	14.74

Supplemental Table 2. HTNV Gn 15-mer peptides with an 8-amino acid overlap.

GN-1	LRNVYDMKIECPHTV	GN-46	AKHVFSPGLFPKLNH
GN-2	KIECPHTVSFGENSV	GN-47	GLFPKLNHTNCDKSA
GN-3	VSFGENSVIGYVELP	GN-48	HTNCDKSAIPLIWTG
GN-4	VIGYVELPPVPLADT	GN-49	AIPLIWTGMIDLPGY
GN-5	PPVPLADTAQMVPES	GN-50	GMIDLPGYYEAVHPC
GN-6	TAQMVPESSCNMDNH	GN-51	YYEAVHPCTVFCVLS
GN-7	SSCNMDNHQSLNTIT	GN-52	CTVFCVLSGPGASCE
GN-8	HQSLNTITKYTQVSW	GN-53	SGPGASCEAFSEGGI
GN-9	TKYTQVSWRGKADQS	GN-54	EAFSEGGIFNITSPM
GN-10	WRGKADQSQSSQNSF	GN-55	IFNITSPMCLVSKQN
GN-11	SQSSQNSFETVSTEV	GN-56	MCLVSKQNRFRLTEQ
GN-12	FETVSTEVDLKGTCV	GN-57	NRFRLTEQQVNFVCQ
GN-13	VDLKGTCVLKHKMVE	GN-58	QQVNFVCQRVDMDIV
GN-14	VLKHKMVEESYRSRK	GN-59	QRVDMDIVVYCNGQR
GN-15	EESYRSRKSVTCYDL	GN-60	VVYCNGQRKVILTKT
GN-16	KSVTCYDLSCNSTYC	GN-61	RKVILTKTLVIGQCI
GN-17	LSCNSTYCKPTLYMI	* GN-62	TLVIGQCIYTITSLF
GN-18	CKPTLYMIVPIHACN	GN-63	IYTITSLFSLLPGVA
GN-19	IVPIHACNMMKSCLI	GN-64	FSLLPGVAHSIAVEL
GN-20	NMMKSCLIALGPYRV	GN-65	AHSIAVELCVPGFHG
GN-21	IALGPYRVQVVYERS	GN-66	LCVPGFHGWATAALL
GN-22	VQVVYERSYCMTGVL	* GN-67	GWATAALLVTFCFGW
GN-23	SYCMTGVLIEGKCFV	GN-68	LVTFCFGWVLIPAIT
GN-24	LIEGKCFVPDQSVVS	GN-69	WVLIPAITFIILTVL
GN-25	VPDQSVVSIIKHGIF	GN-70	TFIILTVLKFIANIF
GN-26	SIIKHGIFDIASVHI	GN-71	LKFIANIFHTSNQEN
* GN-27	FDIASVHIVCFFVAV	GN-72	FHTSNQENRLKSVLR
GN-28	IVCFFVAVKGNTYKI	GN-73	NRLKSVLRKIKEEFE
GN-29	VKGNTYKIFEQVKKS	GN-74	RKIKEEFEKTKGSMV
* GN-30	IFEQVKKSFESTCND	GN-75	EKTKGSMVCDVCKYE
GN-31	SFESTCNDTENKVQG	GN-76	VCDVCKYECETYKEL
GN-32	DTENKVQGYYICIVG	GN-77	ECETYKELKAHGVSC
* GN-33	GYYICIVGGNSAPIY	GN-78	LKAHGVSCPQSQCPY
GN-34	GGNSAPIYVPTLDDF	GN-79	CPQSQCPYCFTHCEP
GN-35	YVPTLDDFRSMEAFT	GN-80	YCFTHCEPTEAAFQA
GN-36	FRSMEAFTGIFRSPH	GN-81	PTEAAFQAHYKVCQV
GN-37	TGIFRSPHGEDHDLA	GN-82	AHYKVCQVTHRFRDD
GN-38	HGEDHDLAGEEIASY	GN-83	VTHRFRDDLKKTVTP
GN-39	AGEEIASYSIVGPAN	GN-84	DLKKTVTPQNFTPGC
GN-40	YSIVGPANAKVPHSA	GN-85	PQNFTPGCYRTLNLF
GN-41	NAKVPHSASSDTLSL	GN-86	CYRTLNLFRYKSRCY
GN-42	ASSDTLSLIAYSGIP	GN-87	FRYKSRCYIFTMWIF
GN-43	LIAYSGIPSYSSLSI	* GN-88	YIFTMWIFLLVLESI
GN-44	PSYSSLSILTSSTEA	GN-89	FLLVLESILWAASA
GN-45	ILTSSTEAKHVFSPG		

* Failed to synthesize.

Supplemental Table 3. HTNV Gc 15-mer peptides with an 8-amino acid overlap.

GC-1	SETPLTPVWNDNAHG	GC-41	TSTMHFTDERIEWKD
GC-2	VWNDNAHGVGSVPMH	GC-42	DERIEWKDPDGMLRD
GC-3	GVGSVPMHTDLELDF	GC-43	DPDGMLRDHINILVT
GC-4	HTDLELDFSLTSSSK	GC-44	DHINILVTKDIDFDN
GC-5	FSLTSSSKYTYRRKL	GC-45	TKDIDFDNLGENPCK
GC-6	KYTYRRKLTNPLEEA	GC-46	NLGENPCKIGLQTSS
GC-7	LTNPLEEAQSIDLHI	GC-47	KIGLQTSSIEGAWGS
GC-8	AQSIDLHIEIEEQTI	GC-48	SIEGAWGSGVGFTLT
GC-9	IEIEEQTIGVDVHAL	GC-49	SGVGFTLTCLVSLTE
GC-10	IGVDVHALGHWFDGR	GC-50	TCLVSLTECPTFLTS
GC-11	LGHWFDGRLNLKTSF	GC-51	ECPTFLTSIKACDKA
GC-12	RLNLKTSFHCYGACT	GC-52	SIKACDKAICYGAES
GC-13	FHCYGACTKYEYPWH	GC-53	AICYGAESVTLTRGQ
GC-14	TKYEYPWHTAKCHYE	GC-54	SVTLTRGQNTVKVSG
GC-15	HTAKCHYERDYQYET	GC-55	QNTVKVSGKGGHSGS
GC-16	ERDYQYETSWGCNPS	GC-56	GKGGHSGSTFRCCHG
GC-17	TSWGCNPSDCPGVGT	GC-57	STFRCCHGEDCSQIG
GC-18	SDCPGVGTGCTACGL	GC-58	GEDCSQIGLHAAAPH
GC-19	TGCTACGLYLDQLKP	GC-59	GLHAAAPHLDKVNGI
GC-20	LYLDQLKPVGSAYKI	GC-60	HLDKVNGISEIENSK
GC-21	PVGSAYKIITIRYSR	GC-61	ISEIENSKVYDDGAP
GC-22	IITIRYSRRVCVQFG	GC-62	KVYDDGAPQCGIKCW
GC-23	RRVCVQFGEENLCKI	* GC-63	PQCGIKCWFVKSGEW
GC-24	GEENLCKIIDMNDCF	GC-64	WFVKSGEWISGIFSG
GC-25	IIDMNDCFVSRHVKV	* GC-65	WISGIFSGNWIVLIV
GC-26	FVSRHVKVCIIGTVS	* GC-66	GNWIVLIVLCVFLLF
GC-27	VCIIGTVSKFSQGDT	* GC-67	VLCVFLLFSLVLLSI
GC-28	SKFSQGDTLLFFGPL	GC-68	FSLVLLSIL
GC-29	TLLFFGPLEGGGLIF		
GC-30	LEGGGLIFKHWCTST		
GC-31	FKHWCTSTCQFGDPG		
GC-32	TCQFGDPGDIMSPRD		
GC-33	GDIMSPRDKGFLCPE		
GC-34	DKGFLCPEFPGSFRK		
GC-35	EFPGSFRKKCNFATT		
GC-36	KKCNFATTPICEYDG		
GC-37	TPICEYDGNMVSGYK		
GC-38	GNMVSGYKKVMATID		
GC-39	KKVMATIDSFQSFNT		
GC-40	DSFQSFNTSTMHFTD		

* Failed to synthesize.
