# Supplementary material for: Prefusion-stabilized Hantaan virus glycoprotein nucleic acid vaccine elicits potent neutralizing antibody responses via germinal center activation
Source: Nat Commun. 2026 Mar 14;17:3972. doi: 10.1038/s41467-026-70285-7 (PMC13133349; doi:10.1038/s41467-026-70285-7)
Supplement: Supplementary file 3 — Reporting Summary [file 41467_2026_70285_MOESM3_ESM.pdf]

nature portfolio

Corresponding author(s): YE Wei

Last updated by author(s): Feb 10, 2026

Reporting Summary

Nature Portfolio wishes to improve the reproducibility of the work that we publish. This form provides structure for consistency and transparency in reporting. For further information on Nature Portfolio policies, see our Editorial Policies and the Editorial Policy Checklist.

Statistics

For all statistical analyses, confirm that the following items are present in the figure legend, table legend, main text, or Methods section.

n/a	Confirmed	
☐	☒	The exact sample size (*n*) for each experimental group/condition, given as a discrete number and unit of measurement
☐	☒	A statement on whether measurements were taken from distinct samples or whether the same sample was measured repeatedly
☐	☒	The statistical test(s) used AND whether they are one- or two-sided<br>*Only common tests should be described solely by name; describe more complex techniques in the Methods section.*
☐	☒	A description of all covariates tested
☐	☒	A description of any assumptions or corrections, such as tests of normality and adjustment for multiple comparisons
☐	☒	A full description of the statistical parameters including central tendency (e.g. means) or other basic estimates (e.g. regression coefficient) AND variation (e.g. standard deviation) or associated estimates of uncertainty (e.g. confidence intervals)
☐	☒	For null hypothesis testing, the test statistic (e.g. *F*, *t*, *r*) with confidence intervals, effect sizes, degrees of freedom and *P* value noted<br>*Give P values as exact values whenever suitable.*
☒	☐	For Bayesian analysis, information on the choice of priors and Markov chain Monte Carlo settings
☒	☐	For hierarchical and complex designs, identification of the appropriate level for tests and full reporting of outcomes
☒	☐	Estimates of effect sizes (e.g. Cohen's *d*, Pearson's *r*), indicating how they were calculated

Our web collection on statistics for biologists contains articles on many of the points above.

Software and code

Policy information about availability of computer code

Data collection

The following tools are used for data collection:
(1) Beckman coulter CytoFLEX flow cytometer (Beckman);
(2) Olympus BX60 (Olympus);
(3) Olympus IX71 (Olympus);
(4) Olympus OlyVIA 4.1.1 (Olympus);
(5) Odyssey CLx Infrared Imaging System (LI-COR Biosciences);
(6) LightCycler® 96 Application Software (Roche);
(7) Biotek Synergy 2 SL Microplate Reader (Biotek);
(8) AID Classic EliSpot Reader (AID);
(9) Zeiss LSM880 LSCM (Carl Zeiss AG);
(10) BC-5000 Vet Auto Hematology Analyzer (mindray);
(11) BC-30 Vet Auto Hematology Analyzer (mindray);
(12) Panoramic MIDI (3DHISTECH).

Data analysis

The following software were used for data analysis:
(1) Image Studio Lite Software (Odyssey) were used for immunoblot analysis;
(2) FlowJo software (version 10) and CytExpert was used for analysis of FACS data;
(3) GraphPad Prism (version 8.0) and SPSS (version 26.0) were used for statistical analysis;
(4) Image J (version 1.48v) was used for analysis of immunofluorescence data;

For multiple comparisons, two-way analysis of variance (ANOVA) or one-way ANOVA followed by
Tukey's multiple comparisons test. Differences are considered statistically significant when the p values were <0.05 (*), <0.01 (**) and <0.001 (***). Statistically nonsignificant data (p value > 0.05) are indicated as NS or not indicated. Data are presented as the mean ± SEM if not stated otherwise in the figure legends.
The exact p values are shown in the source data file.

For manuscripts utilizing custom algorithms or software that are central to the research but not yet described in published literature, software must be made available to editors and reviewers. We strongly encourage code deposition in a community repository (e.g. GitHub). See the Nature Portfolio guidelines for submitting code & software for further information.

Data

Policy information about availability of data

All manuscripts must include a data availability statement. This statement should provide the following information, where applicable:
- Accession codes, unique identifiers, or web links for publicly available datasets
- A description of any restrictions on data availability
- For clinical datasets or third party data, please ensure that the statement adheres to our policy

All data generated in this study are provided in the Source Data file associated with this paper. No sequencing or other omics data requiring deposition in public repositories were generated. Source data are provided with this paper. All reagents will be made available upon request after the completion of a material transfer agreement. Requests can be directed to the corresponding author.

Research involving human participants, their data, or biological material

Policy information about studies with human participants or human data. See also policy information about sex, gender (identity/presentation), and sexual orientation and race, ethnicity and racism.

Reporting on sex and gender	N/A
Reporting on race, ethnicity, or other socially relevant groupings	N/A
Population characteristics	N/A
Recruitment	N/A
Ethics oversight	N/A

Note that full information on the approval of the study protocol must also be provided in the manuscript.

Field-specific reporting

Please select the one below that is the best fit for your research. If you are not sure, read the appropriate sections before making your selection.

☒ Life sciences ☐ Behavioural & social sciences ☐ Ecological, evolutionary & environmental sciences

For a reference copy of the document with all sections, see nature.com/documents/nr-reporting-summary-flat.pdf

Life sciences study design

All studies must disclose on these points even when the disclosure is negative.

Sample size	This is determined by the software SPSS (power value, more than 0.9), and based on our prior studies and other previous papers with similar experiments. The exact sample size has been displayed in the figures or stated in the figure legend or Method part.
Data exclusions	No data were excluded from the analyses.
Replication	This has been descripbed in the figure legends. For the technical replication, each sample was tested three time for the mean value (Flow cytometry assessment).
Randomization	The samples were randomized acquired through table of random numbers.
Blinding	For the biological experiments, Animals are allocated to their respective group at birth by a blinded investigator. For other experiments, including cell experiments, before performing the corresponding treatment, samples are randomly assigned to control and experimental groups by an investigator blinded to subsequent experimental information using the random number table.

The standard laboratory
procedures are strictly followed to keeping the experimental environment and facilities consistent and
performed under the same conditions.
Investigators are blinded to group allocation during data collection, image quantification and data analysis.

Behavioural & social sciences study design

All studies must disclose on these points even when the disclosure is negative.

Study description	*Briefly describe the study type including whether data are quantitative, qualitative, or mixed-methods (e.g. qualitative cross-sectional, quantitative experimental, mixed-methods case study).*
Research sample	*State the research sample (e.g. Harvard university undergraduates, villagers in rural India) and provide relevant demographic information (e.g. age, sex) and indicate whether the sample is representative. Provide a rationale for the study sample chosen. For studies involving existing datasets, please describe the dataset and source.*
Sampling strategy	*Describe the sampling procedure (e.g. random, snowball, stratified, convenience). Describe the statistical methods that were used to predetermine sample size OR if no sample-size calculation was performed, describe how sample sizes were chosen and provide a rationale for why these sample sizes are sufficient. For qualitative data, please indicate whether data saturation was considered, and what criteria were used to decide that no further sampling was needed.*
Data collection	*Provide details about the data collection procedure, including the instruments or devices used to record the data (e.g. pen and paper, computer, eye tracker, video or audio equipment) whether anyone was present besides the participant(s) and the researcher, and whether the researcher was blind to experimental condition and/or the study hypothesis during data collection.*
Timing	*Indicate the start and stop dates of data collection. If there is a gap between collection periods, state the dates for each sample cohort.*
Data exclusions	*If no data were excluded from the analyses, state so OR if data were excluded, provide the exact number of exclusions and the rationale behind them, indicating whether exclusion criteria were pre-established.*
Non-participation	*State how many participants dropped out/declined participation and the reason(s) given OR provide response rate OR state that no participants dropped out/declined participation.*
Randomization	*If participants were not allocated into experimental groups, state so OR describe how participants were allocated to groups, and if allocation was not random, describe how covariates were controlled.*

Ecological, evolutionary & environmental sciences study design

All studies must disclose on these points even when the disclosure is negative.

Study description	*Briefly describe the study. For quantitative data include treatment factors and interactions, design structure (e.g. factorial, nested, hierarchical), nature and number of experimental units and replicates.*
Research sample	*Describe the research sample (e.g. a group of tagged Passer domesticus, all Stenocereus thurberi within Organ Pipe Cactus National Monument), and provide a rationale for the sample choice. When relevant, describe the organism taxa, source, sex, age range and any manipulations. State what population the sample is meant to represent when applicable. For studies involving existing datasets, describe the data and its source.*
Sampling strategy	*Note the sampling procedure. Describe the statistical methods that were used to predetermine sample size OR if no sample-size calculation was performed, describe how sample sizes were chosen and provide a rationale for why these sample sizes are sufficient.*
Data collection	*Describe the data collection procedure, including who recorded the data and how.*
Timing and spatial scale	*Indicate the start and stop dates of data collection, noting the frequency and periodicity of sampling and providing a rationale for these choices. If there is a gap between collection periods, state the dates for each sample cohort. Specify the spatial scale from which the data are taken*
Data exclusions	*If no data were excluded from the analyses, state so OR if data were excluded, describe the exclusions and the rationale behind them, indicating whether exclusion criteria were pre-established.*
Reproducibility	*Describe the measures taken to verify the reproducibility of experimental findings. For each experiment, note whether any attempts to repeat the experiment failed OR state that all attempts to repeat the experiment were successful.*
Randomization	*Describe how samples/organisms/participants were allocated into groups. If allocation was not random, describe how covariates were controlled. If this is not relevant to your study, explain why.*
Blinding	*Describe the extent of blinding used during data acquisition and analysis. If blinding was not possible, describe why OR explain why blinding was not relevant to your study.*

Did the study involve field work? ☐ Yes ☐ No

Field work, collection and transport

Field conditions	*Describe the study conditions for field work, providing relevant parameters (e.g. temperature, rainfall).*
Location	*State the location of the sampling or experiment, providing relevant parameters (e.g. latitude and longitude, elevation, water depth).*
Access & import/export	*Describe the efforts you have made to access habitats and to collect and import/export your samples in a responsible manner and in compliance with local, national and international laws, noting any permits that were obtained (give the name of the issuing authority, the date of issue, and any identifying information).*
Disturbance	*Describe any disturbance caused by the study and how it was minimized.*

Reporting for specific materials, systems and methods

We require information from authors about some types of materials, experimental systems and methods used in many studies. Here, indicate whether each material, system or method listed is relevant to your study. If you are not sure if a list item applies to your research, read the appropriate section before selecting a response.

Materials & experimental systems

n/a	Involved in the study	
☐	☒	Antibodies
☐	☒	Eukaryotic cell lines
☒	☐	Palaeontology and archaeology
☐	☒	Animals and other organisms
☒	☐	Clinical data
☒	☐	Dual use research of concern
☒	☐	Plants

Methods

n/a	Involved in the study	
☒	☐	ChIP-seq
☐	☒	Flow cytometry
☒	☐	MRI-based neuroimaging

Antibodies

Antibodies used

For flow cytometry assays,APC-Cyanine7 Anti-Mouse CD185 (CXCR5) (Biolegend; Cat# 145526;
RRID: AB_25566799) ; APC-Fire™750
Anti-Mouse CD8 (Biolegend; Cat# 140420; RRID: AB_2819885) ;
PE-Cyanine7 Anti-Mouse CD3 (Biolegend; Cat# 100220; RRID:
AB_1732057) ; FITC Anti-Mouse CD4 (Biolegend; Cat# 100406; RRID: AB_312691);
APC-Cyanine7 Anti-Mouse CD45R/B220
(Biolegend; Cat# 103224; RRID: AB_313007) ; FITC Anti-Mouse CD19 (Biolegend; Cat# 152404;
RRID: AB_2629813) ; APC-Fire™750
Anti-Mouse CD38 (Biolegend; Cat# 102738; RRID: AB_2876402);
PE-Cyanine7 Anti-Mouse CD138 (Biolegend; Cat# 112506; RRID:
AB_3674972); PerCP-Cyanine5.5 Anti-Mouse CD95 (Biolegend; Cat# 152610; RRID: AB_2632905) ;
APC Anti-Mouse IFN-γ (Biolegend;
Cat# 505810; RRID: AB_315404); PE Anti-Mouse IL-4 (Biolegend; Cat# 504104; RRID: AB_315318) ;
PerCP-eFluor™ 710 TNF-α
(eBioscience™;Cat#46-7321-82;RRID:AB_1834445).
For flow cytometry, antibodies are added 1 μL per 10^6 cells.
For immunoblot&immunofluorescent measurements,
Anti-Myc Tag Antibody [9E10] (Abcam; Cat# ab32; RRID: AB_303599) ; Mouse
monoclonal Anti-HTNV NP (1A8) (Prepared by our Lab);
Mouse monoclonal Anti-HTNV GP (3G1) (Prepared by our Lab); IRDye®
680CW Goat Anti-Mouse IgG (H + L) (LI-COR; Cat #925-68070; RRID: AB_2651128);
HRP-conjugated Goat Anti-Mouse IgG (Cat#
C650201). For immunoblot, antibodies are diluted as 1:1000;
for immunostaining, antibodies are diluted as 1:200.

Validation

All the antibodies have been confirmed with immunoblot or immunofluorescent assays,
which is marked with the RRID number as
shown above. Related data could also be found on the website of associated companies.

Eukaryotic cell lines

Policy information about cell lines and Sex and Gender in Research

Cell line source(s)	HEK 293T were obtained from Clontech(https://www.takarabio.com/);<br>Vero E6 cells were obtained from ATCC (https://www.atcc.org/).<br>Huh7 (Procell, Wuhan, China; CL-0120)
Authentication	Authentication of the cell line were performed by a Human STR Profiling Cell Authentication Service
Mycoplasma contamination	Cells tested negative for mycoplasma contamination.
Commonly misidentified lines<br>(See ICLAC register)	No misidentified lines were used in the study.

Palaeontology and Archaeology

Specimen provenance	*Provide provenance information for specimens and describe permits that were obtained for the work (including the name of the issuing authority, the date of issue, and any identifying information). Permits should encompass collection and, where applicable, export.*
Specimen deposition	*Indicate where the specimens have been deposited to permit free access by other researchers.*
Dating methods	*If new dates are provided, describe how they were obtained (e.g. collection, storage, sample pretreatment and measurement), where they were obtained (i.e. lab name), the calibration program and the protocol for quality assurance OR state that no new dates are provided.*

☐ Tick this box to confirm that the raw and calibrated dates are available in the paper or in Supplementary Information.

Ethics oversight	*Identify the organization(s) that approved or provided guidance on the study protocol, OR state that no ethical approval or guidance was required and explain why not.*

Note that full information on the approval of the study protocol must also be provided in the manuscript.

Animals and other research organisms

Policy information about studies involving animals; ARRIVE guidelines recommended for reporting animal research, and Sex and Gender in Research

Laboratory animals	The BALB/c mice (six- to eight-week-old male adult mice weighing 20-22 g) were provided by the<br>Experimental Animal Center of<br>Fourth Military Medical University. All animals were housed in standard cages in a temperature- and<br>humidity-controlled<br>environment on a 12-h light/dark cycle (temperature: 23 ±1 °C; relative humidity: 50–60%)<br>with free access to water. Mice were<br>euthanized by CO_2 inhalation at the appropriate time during the study and tissue samples were<br>removed for further experiments.
Wild animals	N/A
Reporting on sex	Only the female mice were enrolled.
Field-collected samples	No field collected samples were used in the study.
Ethics oversight	The experiments were performed in accordance with the recommendations of the Guide for the Care and<br>Use of Laboratory Animals of the Ministry of Science and Technology of China. The protocols were approved<br>by the Animal Ethics Committee of the Air Force Medical University<br>(Fourth Military Medical University; approval no. FMMU-20200403).

Note that full information on the approval of the study protocol must also be provided in the manuscript.

Clinical data

Policy information about clinical studies

All manuscripts should comply with the ICMJE guidelines for publication of clinical research and a completed CONSORT checklist must be included with all submissions.

Clinical trial registration	*Provide the trial registration number from ClinicalTrials.gov or an equivalent agency.*
Study protocol	*Note where the full trial protocol can be accessed OR if not available, explain why.*

Data collection	*Describe the settings and locales of data collection, noting the time periods of recruitment and data collection.*
Outcomes	*Describe how you pre-defined primary and secondary outcome measures and how you assessed these measures.*

Dual use research of concern

Policy information about dual use research of concern

Hazards

Could the accidental, deliberate or reckless misuse of agents or technologies generated in the work, or the application of information presented in the manuscript, pose a threat to:

No	Yes	
☐	☐	Public health
☐	☐	National security
☐	☐	Crops and/or livestock
☐	☐	Ecosystems
☐	☐	Any other significant area

Experiments of concern

Does the work involve any of these experiments of concern:

No	Yes	
☐	☐	Demonstrate how to render a vaccine ineffective
☐	☐	Confer resistance to therapeutically useful antibiotics or antiviral agents
☐	☐	Enhance the virulence of a pathogen or render a nonpathogen virulent
☐	☐	Increase transmissibility of a pathogen
☐	☐	Alter the host range of a pathogen
☐	☐	Enable evasion of diagnostic/detection modalities
☐	☐	Enable the weaponization of a biological agent or toxin
☐	☐	Any other potentially harmful combination of experiments and agents

Plants

Seed stocks	N/A
Novel plant genotypes	N/A
Authentication	N/A

ChIP-seq

Data deposition

☐ Confirm that both raw and final processed data have been deposited in a public database such as GEO.

☐ Confirm that you have deposited or provided access to graph files (e.g. BED files) for the called peaks.

Data access links<br>*May remain private before publication.*	*For "Initial submission" or "Revised version" documents, provide reviewer access links. For your "Final submission" document, provide a link to the deposited data.*
Files in database submission	*Provide a list of all files available in the database submission.*
Genome browser session<br>(e.g. UCSC)	*Provide a link to an anonymized genome browser session for "Initial submission" and "Revised version" documents only, to enable peer review. Write "no longer applicable" for "Final submission" documents.*

Methodology

Replicates	*Describe the experimental replicates, specifying number, type and replicate agreement.*
Sequencing depth	*Describe the sequencing depth for each experiment, providing the total number of reads, uniquely mapped reads, length of reads and whether they were paired- or single-end.*
Antibodies	*Describe the antibodies used for the ChIP-seq experiments; as applicable, provide supplier name, catalog number, clone name, and lot number.*
Peak calling parameters	*Specify the command line program and parameters used for read mapping and peak calling, including the ChIP, control and index files used.*
Data quality	*Describe the methods used to ensure data quality in full detail, including how many peaks are at FDR 5% and above 5-fold enrichment.*
Software	*Describe the software used to collect and analyze the ChIP-seq data. For custom code that has been deposited into a community repository, provide accession details.*

Flow Cytometry

Plots

Confirm that:

- [x] The axis labels state the marker and fluorochrome used (e.g. CD4-FITC).
- [x] The axis scales are clearly visible. Include numbers along axes only for bottom left plot of group (a 'group' is an analysis of identical markers).
- [x] All plots are contour plots with outliers or pseudocolor plots.
- [x] A numerical value for number of cells or percentage (with statistics) is provided.

Methodology

Sample preparation	Spleens or lymph nodes were harvested from immunized mice, gently ground, and filtered through a 70-micron cell strainer to obtain single-cell suspensions of spleen or lymphoid tissue. For spleen cell suspensions, red blood cells were lysed using red blood cell lysis buffer (Gibco). Subsequently, stimulate the cell suspensions with HTNV GP peptide fragments. Incubate at 37°C in a 5% CO_2 incubator for 2–4 hours, then add cytokine secretion inhibitors before subsequent cytokine production assays. During flow cytometry analysis, cells are processed in flow cytometry buffer containing 2% fetal bovine serum (Gibco) and 2 mM EDTA in PBS.
Instrument	Samples were analyzed with a BD FACSCalibur™ 3-laser flow cytometer or BD FACSCanto 10-laser flow cytometer (cell number=15,0000/ group). Finally, the data were processed with FlowJo v10 (TreeStar).
Software	FlowJo v10 (TreeStar) and CytExpert 2.4
Cell population abundance	No cell sorting was performed.
Gating strategy	Single cells were selected by FSC/SSC gates and then FSC/FSC-width, and cells were gated to exclude dead cells, doublets and triplets.

- [x] Tick this box to confirm that a figure exemplifying the gating strategy is provided in the Supplementary Information.

Magnetic resonance imaging

Experimental design

Design type	*Indicate task or resting state; event-related or block design.*
Design specifications	*Specify the number of blocks, trials or experimental units per session and/or subject, and specify the length of each trial or block (if trials are blocked) and interval between trials.*
Behavioral performance measures	*State number and/or type of variables recorded (e.g. correct button press, response time) and what statistics were used to establish that the subjects were performing the task as expected (e.g. mean, range, and/or standard deviation across subjects).*

Acquisition

Imaging type(s)	*Specify: functional, structural, diffusion, perfusion.*
Field strength	*Specify in Tesla*
Sequence & imaging parameters	*Specify the pulse sequence type (gradient echo, spin echo, etc.), imaging type (EPI, spiral, etc.), field of view, matrix size,*

slice thickness, orientation and TE/TR/flip angle.

Area of acquisition

State whether a whole brain scan was used OR define the area of acquisition, describing how the region was determined.

Diffusion MRI ☐ Used ☐ Not used

Preprocessing

Preprocessing software

Provide detail on software version and revision number and on specific parameters (model/functions, brain extraction, segmentation, smoothing kernel size, etc.).

Normalization

If data were normalized/standardized, describe the approach(es): specify linear or non-linear and define image types used for transformation OR indicate that data were not normalized and explain rationale for lack of normalization.

Normalization template

Describe the template used for normalization/transformation, specifying subject space or group standardized space (e.g. original Talairach, MNI305, ICBM152) OR indicate that the data were not normalized.

Noise and artifact removal

Describe your procedure(s) for artifact and structured noise removal, specifying motion parameters, tissue signals and physiological signals (heart rate, respiration).

Volume censoring

Define your software and/or method and criteria for volume censoring, and state the extent of such censoring.

Statistical modeling & inference

Model type and settings

Specify type (mass univariate, multivariate, RSA, predictive, etc.) and describe essential details of the model at the first and second levels (e.g. fixed, random or mixed effects; drift or auto-correlation).

Effect(s) tested

Define precise effect in terms of the task or stimulus conditions instead of psychological concepts and indicate whether ANOVA or factorial designs were used.

Specify type of analysis: ☐ Whole brain ☐ ROI-based ☐ Both

Statistic type for inference

(See Eklund et al. 2016)

Specify voxel-wise or cluster-wise and report all relevant parameters for cluster-wise methods.

Correction

Describe the type of correction and how it is obtained for multiple comparisons (e.g. FWE, FDR, permutation or Monte Carlo).

Models & analysis

n/a	Involved in the study
☐	☐ Functional and/or effective connectivity
☐	☐ Graph analysis
☐	☐ Multivariate modeling or predictive analysis

Functional and/or effective connectivity

Report the measures of dependence used and the model details (e.g. Pearson correlation, partial correlation, mutual information).

Graph analysis

Report the dependent variable and connectivity measure, specifying weighted graph or binarized graph, subject- or group-level, and the global and/or node summaries used (e.g. clustering coefficient, efficiency, etc.).

Multivariate modeling and predictive analysis

Specify independent variables, features extraction and dimension reduction, model, training and evaluation metrics.
